# Supplementary material for: Comprehensive Analysis of SnRK Gene Family and their Responses to Salt Stress in Eucalyptus grandis
Source: Int J Mol Sci. 2019 Jun 6;20(11):2786. doi: 10.3390/ijms20112786 (PMC6600528; doi:10.3390/ijms20112786)
Supplement: Supplementary file 1 [file ijms-20-02786-s001.zip › Supplementary Figure.docx]

Supplementary Material

**Comprehensive analysis of SnRK gene family and their responses to salt stress in Eucalyptus grandis**

**Yujiao Wang^1, 2^, Huifang Yan^1^, Zhenfei Qiu^1^, Bing Hu^1^, Bingshan Zeng^1^, Chonglu Zhong^1^, Chunjie Fan^1*^**

*** Correspondence:** Chunjie Fan: [fanchunjie@caf.ac.cn](mailto:fanchunjie@caf.ac.cn)

# Supplementary Figure 1


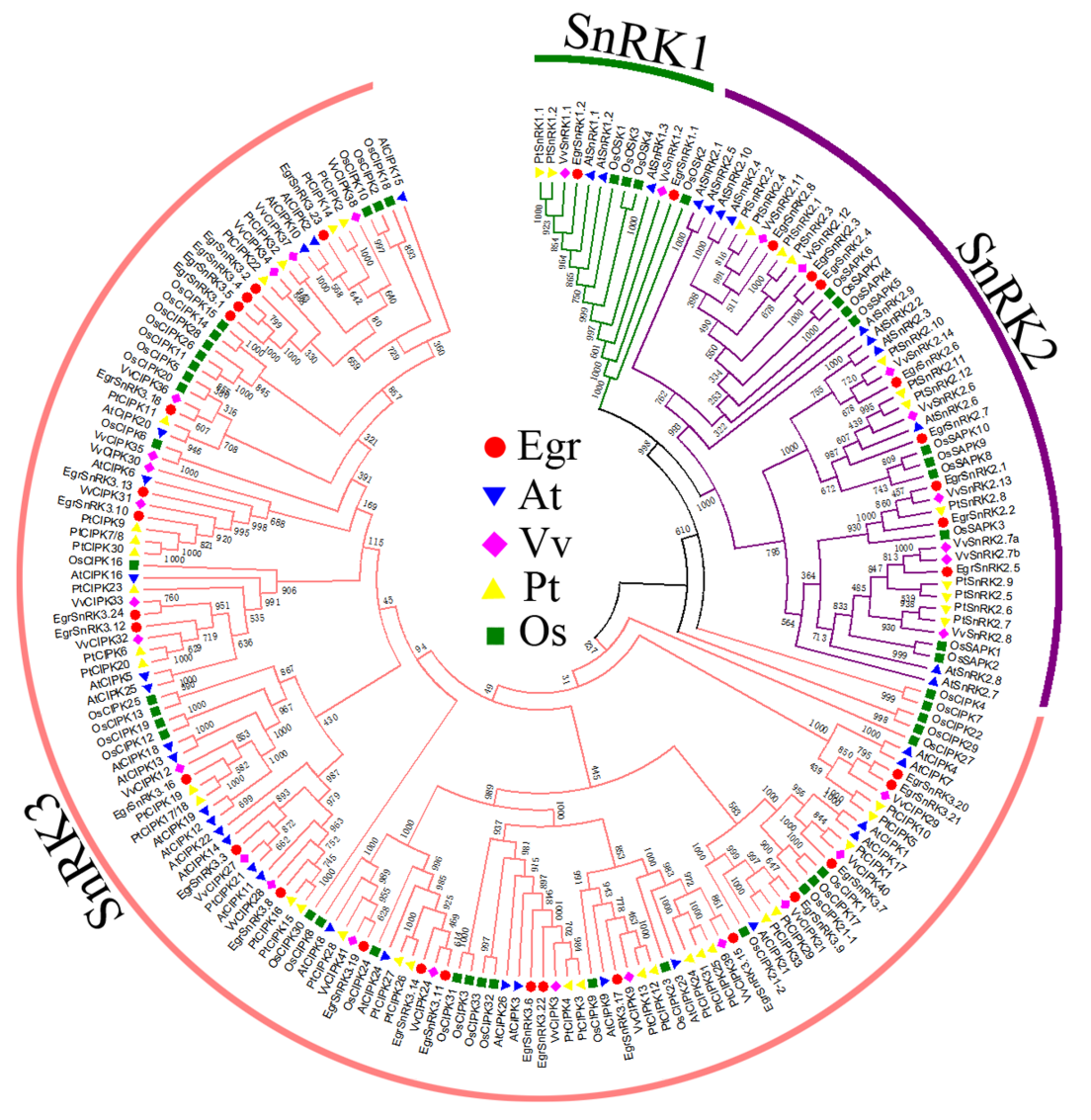


**Supplementary Figure 1. Phylogenetic tree of SnRK genes from *Eucalyptus grandis*, Arabidopsis, rice, grapevine and *populous trichocarpa*. Thirty-four34 EgrSnRK genes, 39 AtSnRK genes, 48 OsSnRK genes, 45 PtSnRK genes and 30 VvSnRK genes are clustered into three3 subgroups (SnRK1, SnRK2 and SnRK3). The SnRK genes from *Eucalyptus grandis*, Arabidopsis, rice, grapevine and *populous trichocarpa* are denoted by red, blue, green, purple and yellow shape, respectively. Details of the SnRK genes from all five plant species are listed in Table S5. The tree was generated using Clustal X 2.0 software using the neighbor-joining (N-J) method.**

# Supplementary Figure 2


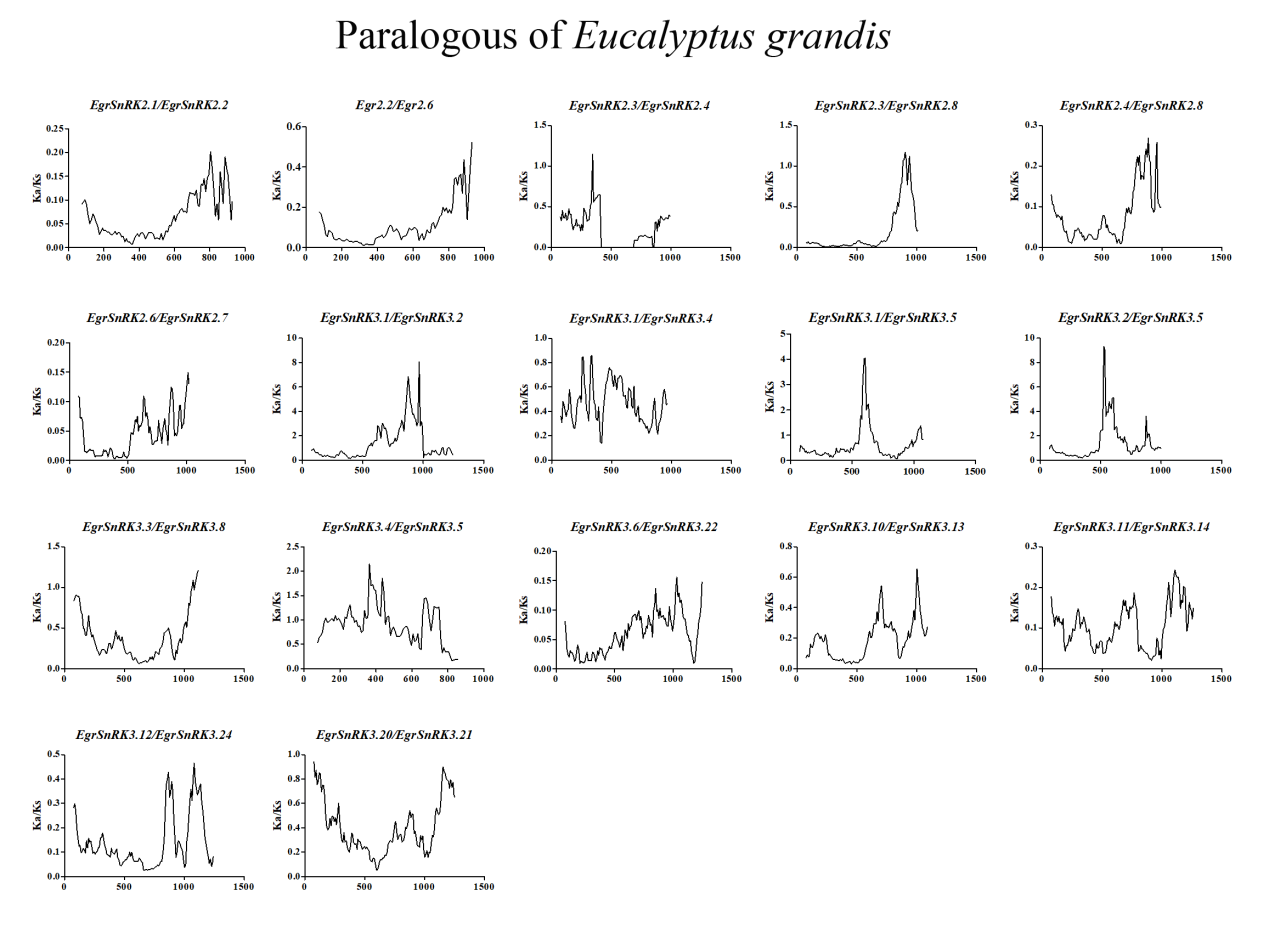


**Supplementary Figure 2. Sliding-window analysis of 17 paralogous gene pairs.**

**The window size is 150 bp, and the step size is 9 bp.**

# Supplementary Figure 3

**
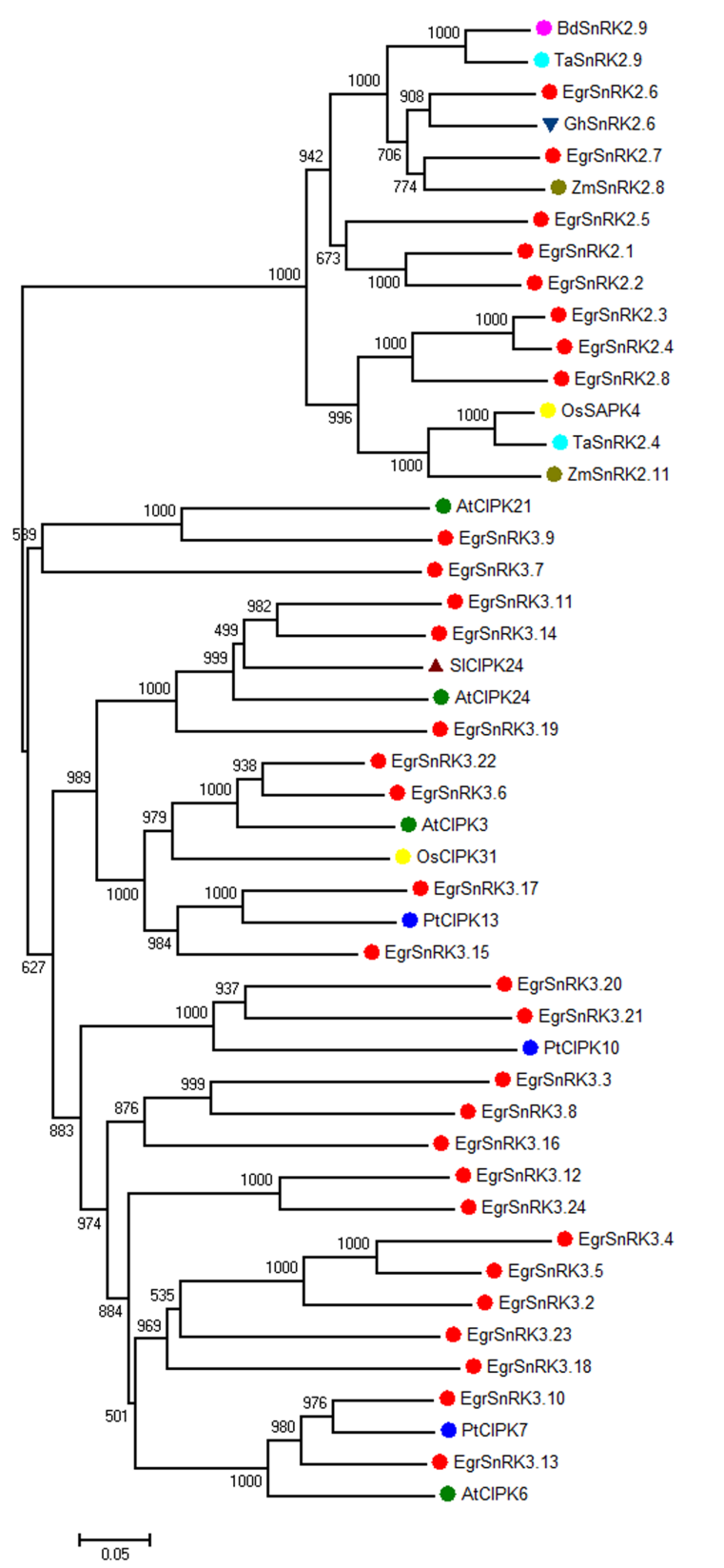
**

**Supplementary Figure 3. Phylogenetic tree of *SnRK* genes from eight species.**

**Egr: *Eucalyptus grandis*, SI: *Solanum lycopersicum L*. Os: *Oryza sativa*, Pt: *populous trichocarpa*, At: *Arabidopsis thaliana*, Zm: *Zea mays*, Gh: *Gossypium hirsutum L.* Bd: *Brachypodium distachyon***
